# Dr. Alexander E. MacDonald

**Published:** 1907-01

**Authors:** 


Dr. Alexander E. MacDonald, of New York, the well known alienist and for a long time superintendent of the Manhattan State Hospital, died at his residence, of tuberculosis, December 7, 1906, aged 61 years.

